# Photosynthetic decline in aging perennial grass is not fully explained by leaf nitrogen

**DOI:** 10.1093/jxb/erac382

**Published:** 2022-10-04

**Authors:** Mauricio Tejera, Nicholas N Boersma, Sotirios V Archontoulis, Fernando E Miguez, Andy VanLoocke, Emily A Heaton

**Affiliations:** Great Lakes Bioenergy Research Center, Michigan State University, East Lansing, MI, USA; Department of Agronomy, Iowa State University, Ames, IA, USA; Department of Agronomy, Iowa State University, Ames, IA, USA; Center for Advanced Bioenergy and Bioproducts Innovation, Urbana, IL, USA; Department of Agronomy, Iowa State University, Ames, IA, USA; Department of Agronomy, Iowa State University, Ames, IA, USA; Department of Agronomy, Iowa State University, Ames, IA, USA; Center for Advanced Bioenergy and Bioproducts Innovation, Urbana, IL, USA; Department of Agronomy, Iowa State University, Ames, IA, USA; Center for Advanced Bioenergy and Bioproducts Innovation, Urbana, IL, USA; Department of Crop Sciences, University of Illinois, Urbana-Champaign, IL, USA; Lancaster University, UK

**Keywords:** Bioenergy, C_4_ metabolism, nitrogen dilution, photosynthesis, plant aging, sink limitation, size effect

## Abstract

Aging in perennial plants is traditionally observed in terms of changes in end-of-season biomass; however, the driving phenological and physiological changes are poorly understood. We found that 3-year-old (mature) stands of the perennial grass *Miscanthus×giganteus* had 19–30% lower *A*_net_ than 1-year-old *M.×giganteus* (juvenile) stands; 10–34% lower maximum carboxylation rates of Rubisco and 34% lower light-saturated *A*_net_ (*A*_sat_). These changes could be related to nitrogen (N) limitations, as mature plants were larger and had 14–34% lower leaf N on an area basis (N_a_) than juveniles. However, N fertilization restored N_a_ to juvenile levels but compensated only 50% of the observed decline in leaf photosynthesis with age. Comparison of leaf photosynthesis per unit of leaf N (PNUE) showed that mature stands had at least 26% lower PNUE than juvenile stands across all N fertilization rates, suggesting that other factors, besides N, may be limiting photosynthesis in mature stands. We hypothesize that sink limitations in mature stands could be causing feedback inhibition of photosynthesis which is associated with the age-related decline in photosynthesis.

## Teaser

Leaf nitrogen content did not fully explain a 10–34% decrease in leaf photosynthesis in aging *Miscanthus×giganteus*. Our data imply that lack of strong carbohydrate sinks late in the season may drive leaf-level photosynthetic decline.

## Introduction

**Fig. 1.**
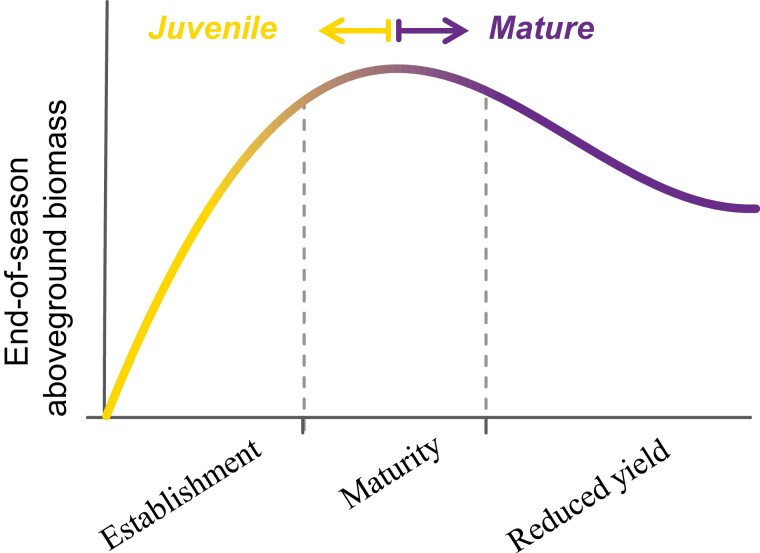
Schematic representation of the growth cycle of a perennial grass in terms of end-of-season above-ground biomass. As the plant ages, perennial plants undergo an establishment phase where end-of-season biomass increases, a maturity phase when plants reach peak yield, and a reduced yield phase associated with plant aging. These changes are also associated with physiological and phenological changes (from yellow to purple in the graph) where juvenile plants present higher leaf photosynthesis, delayed whole-plant senescence, and reduced reproductive allocation, than mature stands. The duration of each phase will depend on species and environment. See text for supporting references.

Aging in perennial plants is traditionally observed in terms of changes in end-of-season biomass. Perennial plants show a common growth pattern over growing seasons; an establishment phase where end-of-season biomass increases, a maturity phase when plants reach peak end-of-season biomass, and a reduced-yield phase associated with plant aging (Fig. 1). While this age response is well conserved across a wide range of perennial species [forest trees (Ryan *et al*., 1997, 2004; Bond, 2000), C_4_ (e.g. Christian *et al.*, 2008; Ramburan *et al.*, 2013; Arundale *et al.*, 2014) and C_3_ (e.g. Angelini *et al.*, 2009) perennial grasses, grasslands (Radrizzani *et al.*, 2010; Jungers *et al.*, 2015), and others (Howeler, 1991; Mantineo *et al.*, 2009)], it is merely observational and does not indicate whether these changes in end-of-season biomass are driven by micro-environmental conditions that change along with the age of the stand (e.g. weed pressure, self-shading, or nutrient limitations), or are driven by intrinsic physiological changes associated with each phase. Understanding the physiological changes associated with each phase would help understand the nature of plant aging and is key to predicting when these phases are likely to occur, as well as what management or genetic interventions can be used to influence their timing.

Similar to plant biomass, leaf photosynthesis also changes in response to whole-plant age. Juvenile plants (i.e. those in the establishment phase) tend to have higher photosynthetic rates than mature individuals that are in the peak- or reducing-yield phase. This age-related decline has been observed in several tree species (Niinemets, 2002; Tang *et al.*, 2014; Chondrogiannis and Grammatikopoulos, 2016) and in warm- and cool-season perennial grasses (Boersma *et al.*, 2015; Jaikumar *et al.*, 2016). As photosynthesis represents the source of carbohydrates for plant growth (i.e. biomass) and metabolism, an age-related decline in photosynthesis could be a potential mechanism behind the observed decline in biomass. However, since leaf photosynthesis is highly dependent on environmental signals as well as internal regulation, it may be that variable growth conditions cause limitations that manifest over time, thus presenting as age effects.

Because plants grow larger as they age, changes in plant size (represented as plant biomass) could also underpin observed age-related changes in leaf photosynthesis. If plants have finite access to nutrients, then nutrient pools inside older, larger individuals would be diluted compared with their younger, smaller counterparts. For example, when given the same amount of soil-available nitrogen (N), older plants must allocate this N to a larger volume of biomass, which manifests as a reduction in the amount of N present per unit of biomass or area. If less N is available in leaves for allocation to photosynthetic enzymes, then observed age effects could be a mere consequence of N limitation. However, older plants have larger root systems with which to mine soil N, and larger N pools that can be transferred to new buds to support initial growth in early spring. Spring N mobilization ranges between 23 kg ha^−1^ and 98 kg ha^−1^ have been reported (Leroy *et al.*, 2022), and these could be help mitigate the size-dependent N limitation, but little is known about the effects of N reserves on leaf N content over years. It is also possible that lower photosynthetic rates result from older plants allocating a larger proportion of N to non-photosynthetic functions as they age, but that is also poorly understood.

Because previous research showed that N requirements are larger late in the season when plants are bigger (Zapater *et al.*, 2017), we tested whether this larger N requirements in bigger plants could lead to larger N limitations in older larger plants compared with juvenile smaller plants. Since 50–70% of N is allocated to photosynthetic enzymes (Makino *et al.*, 2003; Evans and Clarke, 2019), we hypothesized that this N limitation in older larger plants could explain the observed age-related decline in photosynthesis. Changes in leaf N concentrations are an indicator of N availability in the plant and have been shown to be tightly correlated with leaf photosynthetic net CO_2_ assimilation (*A*_net_) (Sinclair and Horie, 1989; Kattge *et al.*, 2009; Sage *et al.*, 2013; Walker *et al.*, 2014). Classically, *A*_net_ shows an asymptotic response to leaf N concentration (expressed as mass per unit area; N_a_): it increases linearly up to high N_a_ levels (0.5–1.5 g N m^−2^), then begins to saturate and plateau (Sinclair and Horie, 1989; Kattge *et al.*, 2009; Sage *et al.*, 2013; Walker *et al.*, 2014). The slope of the linear portion of the *A*_net_/N_a_ slope is known as the photosynthetic nitrogen use efficiency (PNUE) and can be used as an indicator to compare *A*_net_ relative to a unit of leaf N.

Key C_4_ enzymes [phosphoenolpyruvate carboxylase (PEPC), pyruvate phosphate dikinase (PPDK)] and Rubisco abundances also increased linearly with N_a_ (Tazoe *et al.*, 2016). Even when not directly measured, the performance of these enzymes and the photosynthetic apparatus can be inferred by measuring *A*_net_ at different internal CO_2_ concentrations (i.e. *C*_i_ curves; Von Caemmerer and Furbank, 2003; Sharkey *et al.*, 2007; Sage *et al.*, 2013) and different incident light levels (i.e. *A*–*Q* curves; Sage *et al.*, 2013; Coe and Lin, 2018). If the potential N limitation in larger and older plants occurs at the leaf level, then the observed decline in *A*_net_ could be a physiological consequence of reduced leaf N limiting photosynthesis rather than changes associated with plant aging. Consequently, N supply becomes a key variable to control in order to accurately assess the nature of aging in perennial grasses. This is especially important in fast-growing perennial plants that can double or triple their size during establishment (Tejera *et al.*, 2019).

In temperate areas of the USA and Europe, the warm-season perennial grass *Miscanthus×giganteus* (Greef et. Deu.; Hodkinson and Renvoize, 2001) has been adopted for biomass production largely because of its high yields (Heaton *et al.*, 2008; Hastings *et al.*, 2009) and low nutrient requirements. Because *M.×giganteus* is a clonally propagated perennial, establishment is relatively more challenging and expensive compared with seeded C_4_ crops such as maize or sorghum, elevating the importance of understanding and accurately estimating yields over a multi-year life cycle (Edmonds *et al.*, 2021). With minimal genetic variability, and therefore minimal genetic×environment interactions, this clonally propagated C_4_ grass could also help elucidate age-related changes in perennial physiology and the interactive effects of N fertilization.

In a previous study, 1-year-old *M.×giganteus* stands had up to four times greater *A*_net_ than 3-year-old stands (Boersma *et al.*, 2015). These results could suggest an aging response in *M.×giganteus*; however, N could have been a limiting factor as 3-year-old stands were notably bigger in size, and leaf N in 1-year-old stands was up to 2.4 times greater than in 3-year-old stands. In a different study, the response of *M.×giganteus* leaf photosynthesis to N fertilization was found to be smaller than that of leaf N: *A*_net_ and *V*_cmax_ were not affected by N fertilization in 4-year-old stands, even when fertilized stands had ~25% higher leaf N content than unfertilized stands, suggesting that factors other than N limited photosynthesis (Wang *et al.*, 2012).

To our knowledge, no previous research has studied the combined effects of age and N fertilization on photosynthesis in perennial crops. Usually, age-related changes are studied from an ecological perspective on unfertilized co-existing stands of different ages. While representative of natural conditions, this approach may confound micro-environmental conditions (e.g. saplings grown in the understorey and mature trees with leaves in the upper canopy) or different site histories. We posit that these confounding effects have masked age effect differences, limiting the scope of earlier studies. Confounding environmental conditions are especially important when measuring photosynthetic traits as they rapidly change with the environment. To resolve the confounding effects of growing season and age, we suggest age effects should be studied in independent combinations of age and growing season. These combinations are impossible when studying multiple stand ages in one growing season, or one stand over multiple growing seasons. Staggered-start experiments, however, allow for such comparisons by replicating the planting year, effectively starting a new iteration of the experiment each year, to provide independent combinations of age and growing season (Casler, 1999; Loughin, 2006; Tejera *et al.*, 2019).

To elucidate N limitation in older plants and overcome environmental limitations of previous experiments, we combined a staggered-start design (Tejera *et al.*, 2019) with a wide range of N fertilization rates to study age-related changes in *M.×giganteus* photosynthetic performance in the field. The experiment was carried out during the first 3 years of *M.×giganteus* growth, when the plant transitions from juvenile to mature. We chose this period because previous research showed clear phenological and developmental differences between 1-, 2-, and 3-year old stands (Tejera *et al*., 2019, 2021). Overall, 2- and 3-year-old stands behaved similarly, indicating that they had reached maturity, in contrast to the juvenile 1-year-old stands.

In this experiment, we first tested how aging affects photosynthesis in the perennial grass *M.×giganteus*. Our hypotheses were:

Hypothesis (i) Photosynthesis by age: leaf photosynthesis in 2- and 3-year-old (mature) stands would be lower than in 1-year-old (juvenile) stands, and mature stands would not differ from each other. To test this, we compared *M.×giganteus* photosynthetic performance (specifically, *A*_net_ and *A*–*C*_i_ and *A*–*Q* curve parameters), from unfertilized plots, between stand ages.
Hypothesis (ii) Leaf N by age: mature stands, which would be larger in size, would have lower leaf N than juvenile stands. We tested this by comparing leaf N content, on an area (N_a_) and mass (N_m_) basis, of unfertilized stands, across stand ages.
Hypothesis (iii) Photosynthesis by leaf N: leaf photosynthesis and leaf N would increase with N fertilization regardless of stand ages. We tested this by comparing *M.×giganteus* photosynthetic performance, N_a_, and N_m_, averaged over stand ages, across the increasing levels of N fertilization.
Hypothesis (iv) Age by N fertilization: mature stands with reduced leaf photosynthesis and leaf N would reach juvenile leaf photosynthesis and N levels when fertilized with a high enough N fertilization rate. We tested this by comparing *M.×giganteus* photosynthetic performance from mature fertilized plots with unfertilized juvenile plots and by comparing PNUE across stand ages.

If hypothesis (iv) holds true, and there are no differences, then we would conclude that age-related changes in photosynthesis observed in the literature were probably driven by N limitations associated with reduced N per unit of biomass and aging effects would be minimal. Otherwise, if N fertilization does not arrest the aging effect and juvenile stands have higher PNUE, then we would conclude the nature of this age-related change is likely to be driven by ontogenic changes rather than the environment.

## Materials and methods

### Experimental design

This study was conducted in the field at the Iowa State University Sorenson Farm in central Iowa (42.0132 N, –93.7430 W), USA. *Miscanthus×giganteus* clone ‘Freedom’ (sourced from Repreve Renewables, now AGgrow Tech, Greensboro, NC, USA) was planted in 0.3 m rows at a density of ~11 rhizome m^−2^ using a proprietary mechanized plot planter. The Freedom clone is genetically very similar to both the ‘Illinois’ clone widely used in the USA and the ‘EMI-1’ clone widely used in Europe (Głowacka *et al.*, 2015). The experimental design was a staggered-start randomized split-plot with four blocked replications (*n*=4). Stand ages, implemented as planting years (2015, 2016, and 2017), were the main-plot (24 m×60 m) and N fertilization rates (0, 112, 224, 336, and 448 kg ha^−1^) were the subplot (24 m×12 m). That is, 20 subplots (5 N rates×4 blocks=20) were planted each year during 2015–2017. By 2017, the design resulted in 4 replications×3 ages×5 N rates=60 experimental units (subplots) per site. Planting occurred in May, and N treatments were applied within a month. Fertilizer was applied in subsequent years within a month of crop emergence. Weeds were managed with herbicides when needed. See Tejera *et al.* (2019) for full details of plot management. Soils in the experimental site were classified as typic Endoaquolls; our site was characterized by lower soil N, P, and K, and low organic matter (Tejera *et al.*, 2019). Soil N content in the top 0.15 m ranged from 11 kg ha^−1^ to 92 kg ha^−1^ across all plots, but showed no statistical differences prior to the establishment of the crop.

### Weather data

Historic and current weather data (Table 1) were obtained from the Iowa State University Soil Moisture Network (https://mesonet.agron.iastate.edu/). The weather station (Site ID: BOOI4) was 2.7 km from the experimental site. We considered the growing season to start (*T*_i_) on the day of planting for new stands (juvenile stands) and the date of last hard spring frost for established stands (mature stands). Similarly, the end of the growing season (*T*_f_) was defined as the date of the first hard autumnal frost. We defined a hard frost as when the air temperature stayed below 0 °C for >12 h (Kaiser and Sacks, 2015). Thermal time was measured in growing degree days (GDD, °C d) as:

**Table 1.** Current and historic weather data in central Iowa (42.0132N, –93.743W), USA, for three *Miscanthus×giganteus* growing seasons in a staggered-start experiment.

Growing season	Monthly average temperature (°C)	Total GDD_6_	Total precipitation	Total incident PAR (MJ m^−2^)	Last spring frost ^*b*^	First autumnal frost	Growing season length
		(°C)^*a*^	(mm)				(d)
2015	17.5	2540	840	1863	27/03	20/11	238
2016	18.6	2779	713	1884	03/03	19/11	261
2017	18.9	2561	580	1812	15/03	10/28	227
1986-2013	16	2423	656	1768	---	---	---

^*a*^ Growing degree days (GDD) were calculated using 6 °C as the base temperature for growth (Farrell *et al.*, 2006).

^*b*^ Frost is considered as days with air temperatures below 0 °C for >12 h. Hourly weather data were not available to estimate historic events.


$$\text{GDD}=\;\underset{T\text{i}}{\sum^{T\text{f}}}\left[\frac{T_{\text{max}}+\;T_{\text{min}}}{2}\right]-T_{\text{b}}$$ (1)

Where *T*_max_ and *T*_min_ are daily high and low temperatures, and *T*_b_ is a base growth temperature of 6 °C (Farrell *et al.*, 2006).

### Data collection

#### Gas exchange measurements

Photosynthetic gas exchange and chlorophyll fluorescence were surveyed with a portable infrared gas analyzer (LI-6400xt, Licor®, Lincoln, NE, USA) on the youngest, fully expanded leaf (as indicated by ligule presence) in the sunlit upper canopy (Dohleman and Long, 2009). Measurements were taken every 2 weeks beginning when leaves were big enough to fill the leaf cuvette (early summer), until a killing frost (late autumn). A minimum of two randomly selected plants were measured in each plot, with two different LI-6400xt machines. To minimize diurnal variation, measurements were completed within 2 h of solar noon during periods of clear skies. Within a sampling date, environmental conditions in the leaf cuvette were set to match the ambient CO_2_ concentration, temperature, and photosynthetic photon flux density (PPFD) at the commencement of measuring, then kept constant for all measurements that day. Across all sampling dates, relative humidity inside the cuvette was kept between 45% and 65%. Measurements were recorded once values of *A* and stomatal conductance (*g*_s_) reached steady state, typically within 30–50 s.

The responses of *A* to light (*Q*) and intercellular CO_2_ concentration (*C*_i_) curves were measured every month on intact leaves in the field. For the *A*–*Q* curves, we decreased PPFD from 2000 µmol m^−2^ s^–1^ to 0 µmol m^−2^ s^–1^, while keeping the CO_2_ concentration in the cuvette constant at 400 µmol CO_2_ mol^−1^, in 10 steps (2000, 1500, 1000, 750, 500, 250, 150, 100, 50, and 0 µmol m^−2^ s^1^), and logged *A* measurements once stable. For *A*–*C*_i_ curves, *A* was measured at 10 CO_2_ concentrations inside the cuvette (400, 300, 200, 100, 0, 400, 400, 600, 800, 1000, 1200, and 1500 µmol CO_2_ mol^−1^) in 2015 and eight (400, 300, 200, 100, 50, 400, 400, 600, 1000, and 1200 µmol CO_2_ mol^−1^) in 2016 and 2017 to accommodate the larger plot number. In all cases, PPFD inside the cuvette was matched to ambient values then kept constant for the duration of that day’s measurements. *A*–*Q* curve data were then individually modeled with non-rectangular hyperbola to determine: the light-saturated rate of assimilation (*A*_sat_), the maximum efficiency of light-limited net CO_2_ assimilation (ϕ_CO2,max_) and the rate of dark respiration (*R*_d_) (Dohleman and Long, 2009; Archontoulis and Miguez, 2013). *A*–*C*_i_ data were modeled using a three-parameter asymptotic exponential equation to determine the CO_2_-saturated rate of assimilation (*V*_cmax_) and the compensation point (Γ) when net CO_2_ assimilation is zero. The initial slope of the curve (*C*_i_ <100) was used to estimate the maximum carboxylation capacity of phosphoenolpyruvate (*V*_pmax_; Markelz *et al.*, 2011; Li *et al.*, 2021).

As cuvette conditions were set to match environmental light and temperature conditions, extra variability was introduced when observations were compared across sampling dates. Light conditions should not have caused large variability as the PPFD used in *A*–*C*_i_ curves was at least 1200 µmol m^−2^ s^1^, which represents 70% of a clear-sky PPFD value. In all cases, the *A*_net_ value at ambient CO_2_ concentrations was at least 80% of the light-saturated *A*_net_ values measured in the *A*–*Q* curves. Temperature conditions were more variable (Supplementary Fig. S1A), and to account for this variability we also estimated temperature-corrected *A*_net_ values, both for survey and for *A*–*C*_i_ and *A*–*Q* curves. Temperature corrections were estimated based on the Beta equation (Bernacchi *et al.*, 2003; Archontoulis and Miguez, 2013). Different temperature response functions were fitted for the survey *A*_net_, CO_2_- and light-saturated values. These values were then normalized to the predicted value at 25 °C (Supplementary Fig. S1B).

Modulated chlorophyll fluorescence was simultaneously measured with the other photosynthetic parameters. We estimated PSII efficiency (ϕPSII) as the ratio of absorbed photons used in photochemistry after a saturating light pulse (Genty *et al.*, 1989), according to;


$$\phi \text{PSII}=\frac{{F^{\prime }}_{\text{m}}-F_{\text{s}}}{{F^{\prime }}_{\text{m}}}$$ (2)

Where, *F*_s_ is ‘steady-state’ fluorescence, and *F*ʹ_m_ is the maximal fluorescence during a saturating light flash. Decreasing ϕPSII has been previously correlated to *M.×giganteus* senescence (Boersma *et al.*, 2015).

#### Leaf nitrogen measurements

Immediately after photosynthetic measurements were recorded, the measured leaf lamina was excised and used for specific leaf area (SLA; gDM m^2^) and leaf N content estimations. In 2015 and 2016, SLA for each plot was estimated based on a composite sample of 40 1.6 cm^2^ leaf punches taken from all measured leaves within the plot and dried at 60 °C until constant weight. In 2017, excised lamina were kept at ~5 °C and leaf area was measured in the lab with a flatbed leaf scanner (LI-3100C Area Meter; Licor®) and then dried at 60 °C to constant weight. SLA was then estimated as the ratio of average leaf mass to leaf area of all subsamples. All dried samples were then ground to a powder (<1 mm) and a 3–5 mg subsample was mixed with tungsten trioxide and combusted in an elemental analyzer (Elementar, Ronkonkoma, NY, USA) to determine the concentration of N (N_m_; gN g^−1^). Acetanilide was used as a standard N reference. Leaf N content on an area basis (N_a_; gN m^−2^) was estimated as the product of N_m_×SLA.

#### Data analysis

For this analysis, 1-year-old stands were considered juvenile and 2- and 3-year-old stands mature. This determination is based on previous findings that suggested that the phenology of 1-year-old stands clearly differed from that of 2- and 3-year-old stands, indicating that they were developmentally distinct (Tejera *et al.*, 2021). We began measuring gas exchange in mature stands in June, and in juvenile stands 2 months later, when the leaves of juvenile plants developed sufficient laminar tissue to fill the measurement cuvette. To avoid biased comparisons, we studied differences between juvenile and mature stands only on those sampling dates when all stands were present (co-occurring dates; August–November).

To facilitate data analysis and interpretation, we used subsets of the full dataset to test the different hypotheses:

Hypothesis (i) Photosynthesis by age: we compared photosynthetic parameters (e.g. *A*_net_, *V*_pmax_, Γ, *V*_cmax_, ϕ_CO2,max_, *A*_max_, and ϕPSII) of only unfertilized plots of all planting years within co-occurring dates.
Hypothesis (ii) Leaf N by age: we compared N_a_ of only unfertilized plots of all planting years within co-occurring dates.
Hypothesis (iii) Photosynthesis by leaf N: we compared *A*_net_ and N_a_ of all N rates and planting years averaged over stand ages.
Hypothesis (iv) Age by N fertilization: we compared the photosynthetic parameters and N_a_ of fertilized mature stands to juvenile unfertilized stands. Further, we calculated the PNUE as the ratio of *A*_net_ (PNUE_*A*net_), *V*_cmax_ (PNUE_*V*cmax_), and *A*_sat_ (PNUE_*A*sat_) over N_a_.

We used R statistical software (R Core Team, 2017) for all analyses; the specific models, datasets, and procedures depended on the hypothesis and variables considered. For *A*_net_ and N_a_, we used mixed effect linear models to test the different hypotheses. Stand age, N rate, and sampling date were considered as fixed effects. As in Tejera *et al.* (2019), the full random structure that adjusted best to our experimental design (full model; subplots nested in main plots nested in blocks) had many near-zero variance terms. A simpler random structure, with blocks as a random term, was the one that minimized the number of parameters and maximized goodness of fit [using the Akaike information criterion (AIC), Bayesian information criterion (BIC), and log-likelihood)]. We used lmer() in the lme4 package (Bates *et al.*, 2014) to fit the mixed models, Anova() in the car package (Fox and Weisberg, 2011) for analysis of deviance using type II Wald χ^2^ test, and emmeans() in the emmeans package (Lenth *et al.*, 2018) for mean comparison.

**Fig. 2.**
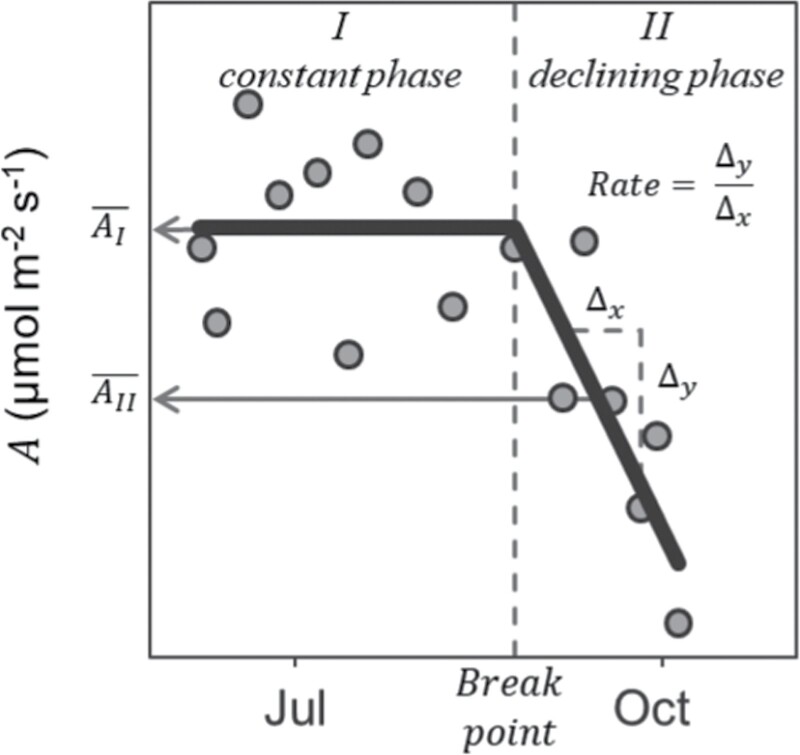
Schematic representation of *M.×giganteus* net CO_2_ assimilation rate (*A*) over the growing season. Two phases were observed in mature stands: (I) a constant phase and (II) a declining phase. We estimated average *A* during the constant phase ($\bar{A_{\text{I}}}$), average *A* during the declining phase ($\bar{A_{\text{II}}}$), rate of decline during the declining phase (Rate), and the day when stands started declining phase (breakpoint), to compare treatment effects. See Fig. 3 for comparison of juvenile and mature rates.

The temporal dynamics of *A*_net_ in mature stands showed two distinct phases; a phase with constant *A* in summer (phase I) followed by steady decline until the first hard frost (phase II; Fig. 2). We compared mature stands based on the average *A*_net_ value of the constant phase ($\bar{A_{\text{I}}}$), the slope (Rate) of *A* during the declining phase $\bar{A_{\text{II}}}$), and the day of the year when the declining phase started (Breakpoint; Fig. 2).

We modeled this dynamic assuming a segmented relationship with time. A segmented relationship is defined by the slope parameters of each line segment, and the breakpoints are where the straight lines connect and the linear relation changes. We used the segmented() function in the segmented package in R (Muggeo, 2008) to model *A* over day of the year and estimate break-points and slope of the two phases. Stand age and N fertilization rates were considered fixed effects for each growing season.

We used non-linear mixed models to model *A*–*C*_i_ and *A*–*Q* data and test stand age and N effects on model parameters. We modeled *A*–*C*_i_ data with an asymptotic exponential three-parameter model (Pinherio and Bates, 2000; Archontoulis and Miguez, 2013):


$$A_{\text{net}}=V_{\text{cmax}}\left(1-e^{-e^{\text{lrc}}\left(C_{\text{i }}-C_{0\;}\right)}\right)$$ (3)

Where net CO_2_ assimilation (*A*_net_) response to internal CO_2_ concentration (*C*_i_) is a function of the maximum rate of Rubisco carboxylation (*V*_max_) and the CO_2_ compensation point (Γ). The lrc parameter is related to the initial slope of the response (*V*_cmax_) but to simplify interpretation we directly estimated *V*_pmax_ as the slope of the *A*–*C*_i_ curve when *C*_i_ is <100 μmol CO_2_. We modeled *A*–*Q* data using a four-parameter non-rectangular model (Johnson *et al.*, 2010; Archontoulis and Miguez, 2013):


$$A_{\text{net}}=\;\frac{A_{\text{sat}}+\;\phi_{{\text{CO}}_{2}}Q-\;\sqrt{{\left(A_{\text{sat}}+\;\;\phi_{{\text{CO}}_{2}}Q\right)}^{2}-4\;\theta \;\phi_{{\text{CO}}_{2}}QA_{\text{sat}}}\;}{2\;\theta \;}-R_{\text{d}}\;$$ (4)

Where net CO_2_ assimilation (*A*_net_) response to light intensity (*Q*) is a function of light-saturated net CO_2_ assimilation (*A*_sat_), the maximum efficiency of light-limited net CO_2_ assimilation (ϕ_CO2_), a curvature parameter (θ), and the *y*-intercept interpreted as leaf respiration in the dark (*R*_d_).

As with linear models, our non-linear model-building strategy was to start with the models that better described our experimental design, with random effects on all curve parameters, and then dropped near-zero variance terms. We chose simpler models based on fewer numbers of parameters and goodness of fit (AIC, BIC, and log-likelihood) (Pinherio and Bates, 2000). Then we included fixed effect terms (i.e. stand age and Nrate) and assessed their effect on the model parameters. We used the nlme R package (Pinherio and Bates, 2000) for model construction and testing, and the emmeans package to compare model parameters.

## Results

**Fig. 3.**
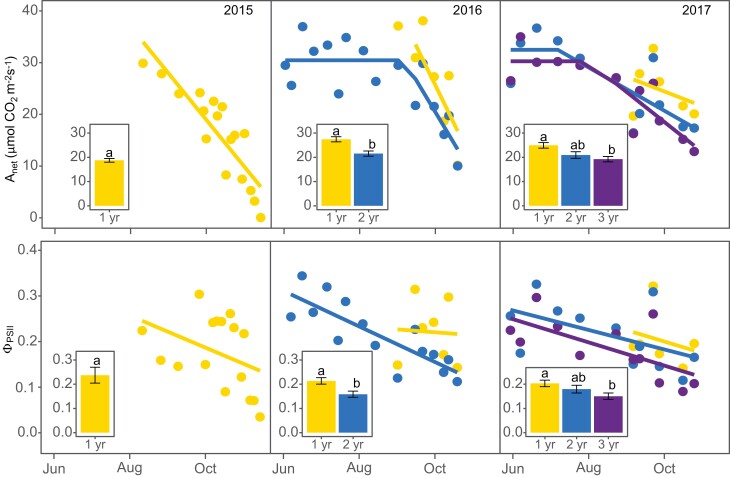
Seasonal changes of *Miscanthus×giganteus* net CO_2_ assimilation (*A*_net_; A) and efficiency of PSII (ϕPSII; B) at different stand ages during three growing seasons. Data correspond to unfertilized plots averaged over four replications. Error bars correspond to ±1 SEM. Inset graphs show average *A*_net_ and ϕPSII over the period of the year when all stand ages occur at the same time. Different letters indicate significant differences between stand ages at *P*-value <0.05

**Fig. 4.**
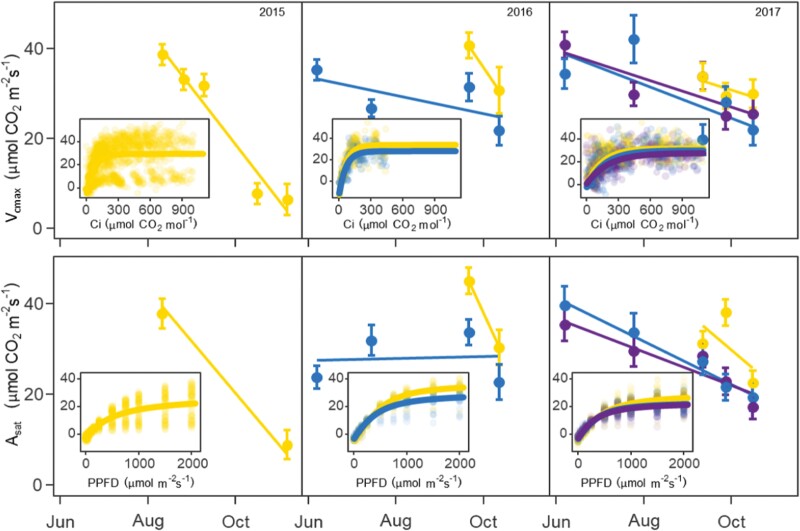
Changes in *Miscanthus×giganteus* maximum carboxylation rate of Rubisco (*V*_cmax_; A) and light-saturated net CO_2_ assimilation (*A*_sat_; B) at different stand ages during the 2015, 2016, and 2017 growing seasons. Data are averages from unfertilized plots (*n*=4). Error bars correspond to ±1 SEM. Inset graphs compile all net CO_2_ assimilation response curves to internal CO_2_ concentration (*C*_i_) and PPFD during the period of the year when all stand ages occurred at the same time.

**Fig. 5.**
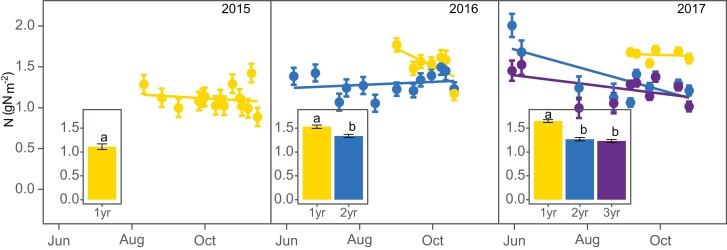
Changes in *Miscanthus×giganteus* area-basis leaf nitrogen content (N_a_) at different stand ages during the 2015, 2016, and 2017 growing seasons. Data are means from unfertilized plots (*n*=4). Error bars correspond to ±1 SEM. Inset graphs show average N_a_ over the period of the year when all stand ages occurred at the same time. Different letters indicate significant differences between stand ages (*P*-value <0.05).

**Fig. 6.**
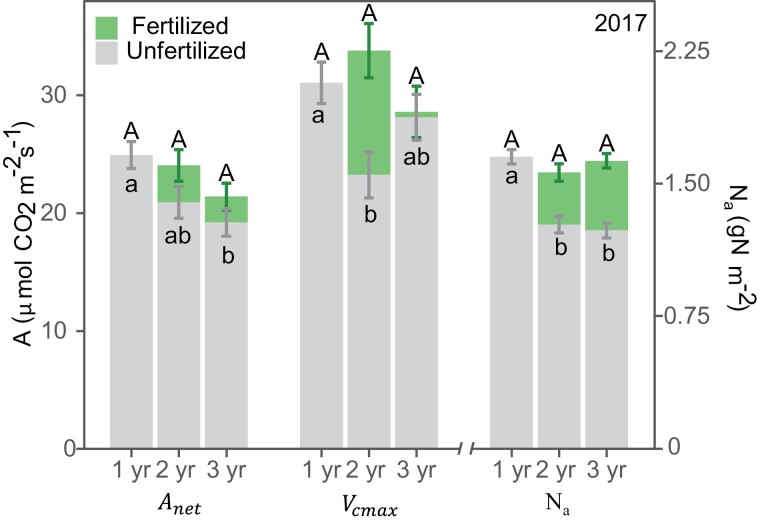
Nitrogen (N) fertilization effect on *Miscanthus×giganteus* age-related changes in leaf photosynthesis [i.e. net CO_2_ assimilation (*A*_net_) and maximum carboxylation rate of Rubisco (*V*_cmax_)], and leaf nitrogen content on an area basis (N_a_) when all stand ages co-occur in 2017 (September–November). See Supplementary Fig. S5 for changes in *A*_sat_ and ϕPSII, and changes in 2016. Different lower case letters indicate significant differences between unfertilized stand ages (gray bars; *P*<0.05). Different upper case letters indicate significant differences between unfertilized juvenile (1-year-old) and fertilized mature (2- and 3-year-old; green bars; *P*<0.05) stands. Values are the average of four replications, and error bars span 2 SEM.

During the first 3 years of growth, the age of the stand was the main driver of differences in *M.×giganteus* photosynthesis and leaf N. Two- and three-year-old (mature) stands had ~20% lower leaf-level photosynthesis (i.e. *A*_net_, *V*_cmax_, and *A*_sat_) and those leaves had 34% lower N_a_ content (Figs 3, 4) than leaves of 1-year-old (juvenile) stands. Nitrogen fertilization increased photosynthetic parameters and N_a_ regardless of the stand age, and *A* and N_a_ leveled off at higher fertilizer rates (Table 2). Fertilized mature stands reached similar photosynthetic and N_a_ values to unfertilized juvenile stands (Figs 5, 6). Nevertheless, when these changes in leaf photosynthesis were analyzed relative to the changes in N_a_, juvenile stands showed higher PNUE across all N rates and higher leaf photosynthesis across the N_a_ range (1.0–2.0 gN m^−2^).

**Table 2.** Nitrogen (N) fertilization effect on *Miscanthus×giganteus* leaf N content on an area basis (N_a_) and leaf photosynthetic parameters [i.e. net CO_2_ assimilation (*A*_net_), PSII efficiency (ϕPSII), and *A*–*C*_i_ and *A*–*Q* response curve parameters].

	Nitrogen fertilization rate (kg N ha^−1^)				
Param	0	112	224	336	448
Survey					
N_a_	1.4 ± 0.055 c^*^	1.6 ± 0.055 b	1.7 ± 0.055 ab	1.8 ± 0.055 a	1.7 ± 0.055 a
*A*_net_	26 ± 0.59 b	28 ± 0.59 ab	28 ± 0.59 a	28 ± 0.59 a	28 ± 0.59 a
ϕPSII	0.2 ± 0.0063	0.21 ± 0.0063	0.21 ± 0.0063	0.21 ± 0.0063	0.21 ± 0.0063
*A*–*C*_i_ curve					
*V*_cmax_	34 ± 1.5 b	36 ± 1.5 ab	41 ± 2 ab	36 ± 1.5 ab	43 ± 2.5 a
lrc	–4.7 ± 0.075 a	–4.8 ± 0.072 a	–5.1 ± 0.078 b	–4.8 ± 0.07 a	–5.2 ± 0.081 b
Γ	5.5 ± 5.2 a	6.7 ± 4.5 a	-20 ± 7.6 b	10 ± 4.2 a	–37 ± 9.9 b
*A*–*Q* curve					
*A*_sat_	29 ± 1.1 b	33 ± 1.1 ab	33 ± 1.1 ab	33 ± 1.1 a	32 ± 1.1 ab
ϕ_CO2_	0.053 ± 0.0013 b	0.061 ± 0.0015 a	0.058 ± 0.0014 ab	0.056 ± 0.0014 ab	0.057 ± 0.0015 ab
θ	0.79 ± 0.021	0.7 ± 0.027	0.75 ± 0.022	0.72 ± 0.026	0.73 ± 0.026
*R*_d_	2.4 ± 0.11 b	3 ± 0.11 a	2.8 ± 0.11 ab	2.8 ± 0.11 ab	2.8 ± 0.11 ab

*A*–*C*_i_ parameters were estimated using a three-parameter asymptotic exponential regression. *A*–*Q* parameters were estimated using a four-parameter non-rectangular hyperbola regression. See the Materials and methods for model equation and parameter interpretation. Values (mean ±SE) are the average over four replicates and all stand ages.

* Different lower case letters indicate significant differences (*P*-value <0.05) between nitrogen rates for each parameter.

### Mature stands had 10–34% lower leaf photosynthesis than juvenile stands

When considering only unfertilized stands, mature stands had 19–30% lower *A*_net_ than juvenile stands in 2016 and 2017 (when measured on co-occurring dates August–November; *P*<0.05; Fig. 3A), and 11–33% lower ΦPSII (*P*<0. 05; Fig. 3B). Similarly, mature stands had 10–34% lower *V*_cmax_ (*P*<0.05; Fig. 4A) and 34% lower *A*_sat_ (*P*<0.05; Fig. 4B). Other A–*C*_i_ and *A*–*Q* parameters showed some trending results but, due to large variability, age and N effects were not significant at 0.05. *A*–*C*_i_ and *A*–*Q* parameters for all sampling dates are presented in Supplementary Tables S1 and S2, respectively.

Mature stands showed a bi-phasic dynamic throughout the growing season of *A*_net_ across ages and years (Fig. 3A). In 2016, the segmented model achieved convergence after two iterations with an *R*^2^=0.65. In 2017, convergence was achieved after nine iterations with an *R*^2^=0.60. Two- and three-year-old stands had very similar *A*_net_ values during the constant phase (*P*>0.05; *A*_2016–2yr_=30.5 ± 1.36 µmol CO_2_ m^−2^ s^−1^; *A*_2017–2yr_=32.5 ± 2.2 µmol CO_2_ m^−2^ s^−1^; *A*_2017–3yr_=30.3 ± 2.95 µmol CO_2_ m^−2^ s^−1^; Fig. 3A), and similar declining rates during the declining phase (*P*>0.05; Rate_2016–2yr_= –0.4 ± 0.15 µmol CO_2_ m^−2^ s^−1^ d^−1^; Rate_2017–2yr_= –0.14 ± 0.056 µmol CO_2_ m^−2^ s^−1^ d^−1^; Rate_2017–3yr_= –0.19 ± 0.081µmol CO_2_ m^−2^ s^−1^ d^−1^; Fig. 3A). *A*_net_ transitioned from the constant to the declining phase on 5 September (± 11 d) in 2016, and on 8 July (± 35 d) and 1 August (± 26 d) for 2-year-old and 3-year-old stands in 2017, respectively.

Temperature-corrected *A*_net_ values did not show the biphasic trend, and steadily declined during the growing season (Supplementary Fig. S2). In spite of the large variability, averaged over growing seasons, the temperature-corrected *A*_net_ rate of decline was 1.5× and 2.5× faster in 2- and 3-year-old stands than in 1-year-old stands. The temperature-corrected *A*_net_ rate of decline over the growing season was the only parameter not different from zero (*P*>0.05). For the other photosynthetic parameters, mature stands had variable responses. Two-year-old stands had 20% lower ΦPSII (Fig. 3B), and 3-year-old stands had 20% higher *V*_cmax_ (Fig. 4A), but none was significantly different (*P*>0.05). Two- and three-year-old stands also had very similar *A*_sat_ values (*P*>0.05; Fig. 4C). Temperature-corrected *V*_cmax_ and *A*_sat_ also declined during the season, but in these cases, none of the slopes was significantly different from zero (*P*>0.05; Supplementary Fig. S3).

### Mature stands had 14–34% lower leaf nitrogen on an area basis than juvenile stands

Unfertilized mature stands had 14–34% lower N_a_ than unfertilized juvenile stands in 2016 and 2017 when measured on co-occurring dates (*P*<0.05; Fig. 5). No differences were found between mature 2- and 3-year-old, stands (*P*>0.05). N_a_ remained almost constant across stand ages during the growing season, especially during the co-occurring period, with a significant but near-zero slope (*P*<0.05; Fig. 5). Similarly, SLA also declined very slightly over the growing season with a slope near zero and very low variance (Supplementary Fig. S4). Juvenile and mature stands had very similar SLA values in all years (*P*>0.05).

### Nitrogen fertilization increased leaf photosynthesis by 8–27% and leaf N by 30% across stand ages

N fertilization increased leaf photosynthesis during the growing season across all stand ages. Compared with the unfertilized stands, the N rate with the largest effect increased *A*_net_ by 8%, *V*_cmax_ by 27%, and *A*_sat_ by 14% (*P*<0.05; Table 2). Similarly, N_a_ increased by 30% in fertilized stands relative to the unfertilized stands averaged over all stand ages (*P*<0.05; Table 2). Along with *A*_sat_, other *A*–*Q* parameters also showed a clear response to N fertilization; ϕ_CO2_ and *R*_d_ increased up to 15% and 25%, respectively. ΦPSII, Γ, and θ were not affected by N fertilization (*P*>0.1).

In spite of the wide range of N fertilization tested in this experiment, leaf photosynthesis showed a rather discrete response to N fertilization, with differences being mainly between no fertilizer and any fertilizer. In the vast majority of cases, the unfertilized treatment was only significantly different at the higher N fertilization rates (i.e. 336 kgN ha^−1^ or 448 kgN ha^−1^), while intermediate N rates did not differ from either high or low extremes (Table 2). In contrast, N_a_ showed an asymptotic response to N fertilization, where the 112 kg N ha^−1^ treatment had significantly higher N_a_ than the unfertilized treatment, and the 336 kgN ha^−1^ or 448 kg N ha^−1^ were also statistically higher than all other treatments. This suggest that both N_a_ and photosynthesis are maximized between 112 kgN ha^−1^ and 224 kg N ha^−1^, since 224 kg N ha^−1^ is not different from 448 kg N ha^−1^ for any parameter.

### Fertilizing mature stands reduced the age decline in leaf photosynthesis by 50% despite similar leaf nitrogen to unfertilized juveniles

N fertilization increased mature stand *A*_net_ by 11–15%, *V*_cmax_ by 2–45%, and *A*_sat_ by 26–34% during 2016 and 2017 when measured on co-occurring dates (*P*-value <0.05; Fig. 6; Supplementary Fig. S5). The ΦPSII response to N fertilization was smaller in mature stands (~ 8–16%). N fertilization of mature stands reduced the magnitude of age-related decline in photosynthetic parameters that had been observed between unfertilized juveniles and mature stands by ~50%. For example, in 2017, unfertilized mature stands had 27% lower *A*_net_ than unfertilized juvenile stands, but this difference dropped to 15% when fertilized mature stands were compared with unfertilized juveniles. In this case, N fertilization reduced the age effect by 56% (15/27=0.56). Overall, the photosynthetic values observed in fertilized mature stands were not statistically different from those seen in unfertilized juvenile stands (*P*>0.05; Fig. 6), and in some cases N fertilization restored photosynthesis values to those seen in juvenile stands.

The fertilization effect on N_a_ was more consistent across years and stand ages; it increased N_a_ by 23–36% (Fig. 6). This increase in N_a_ caused fertilized mature stands to have very similar, or even higher, N_a_ values than unfertilized juvenile stands. Accordingly, mature stands showed a larger N_a_ response to N fertilization than leaf photosynthesis.

**Fig. 7.**
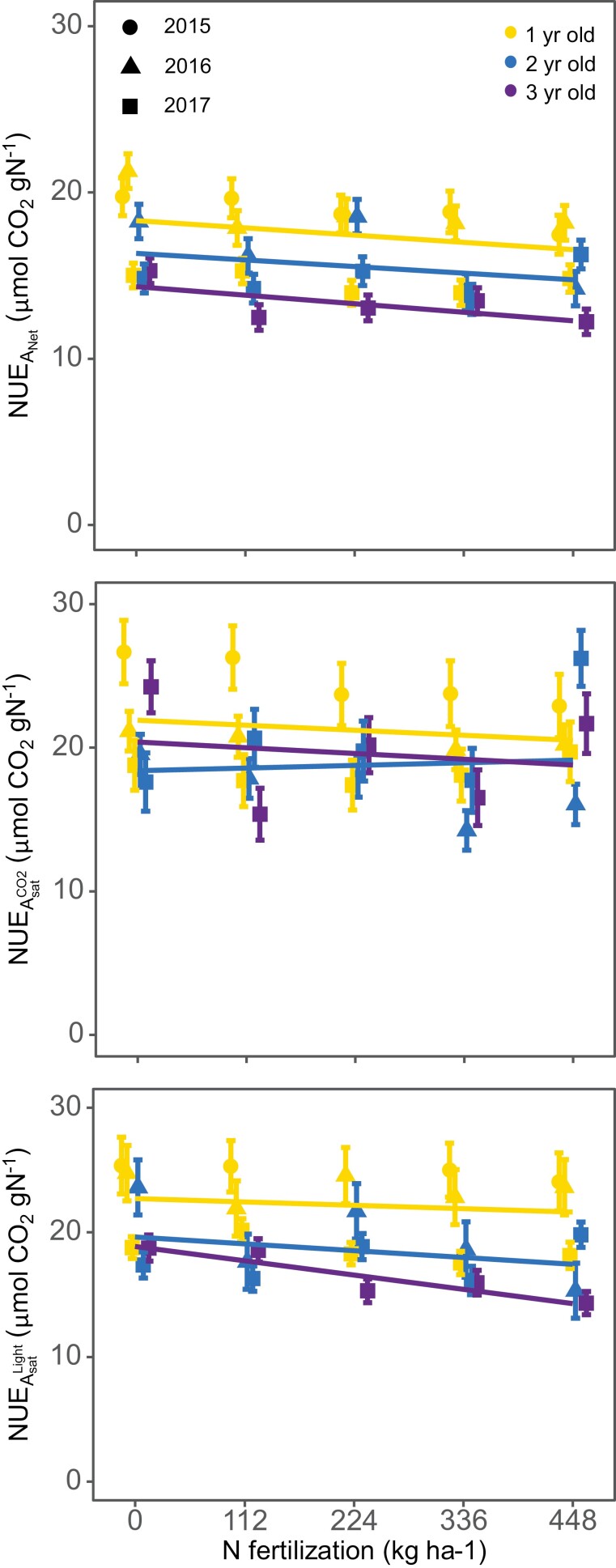
Relationship between nitrogen fertilization and photosynthetic nitrogen use efficiency (expressed on a leaf area basis) in different ages of *Miscanthus×giganteus.* Net CO_2_ assimilation area-based nitrogen use efficiency was estimated using ambient, CO_2_-saturated, and light-saturated net CO_2_ assimilation (top, middle, and center panel, respectively). Data points are averaged over four repetitions. Different growing seasons are indicated with different symbols.

Leaf photosynthesis relative to N_a_ was 35–86% higher in juvenile than in mature stands when estimated based on *A*_net_ (PNUE_*A*net_), and 26–58% higher based on *A*_sat_ (PNUE_*A*sat_) during 2016 and 2017 co-occurring dates, regardless of N fertilization rates (Table 3). The PNUE_*V*cmax_ was rather similar across stand ages; 1-year-old stands had 7% higher PNUE_*V*cmax_ than 2-year-old stands (*P*<0.05), but 3-year-old stands had 7% higher PNUE_*V*cmax_ than 1-year-old stands (*P*<0.05). There was a small, but significant, effect of N fertilization on all NUE indices; all indices showed a slight decline with increasing N fertilization rates (Fig. 7). The age by N fertilization interaction was also significant; however, all NUE indices remained fairly constant across stand ages and N fertilization rates (Fig. 7).

**Table 3.** Changes in *Miscanthus×giganteus* photosynthetic nitrogen use efficiency (μmol CO_2_ s^−1^ gN^−1^), estimated as the ratio of net CO_2_ assimilation (PNUE_*A*net_), maximum carboxylation rate of Rubisco (PNUE_*V*cmax_), and light-saturated net CO_2_ assimilation ( PNUE_*A*sat_) over N_a_ at different stand ages.

	Stand age		
	1 year old	2 year old	3 year old
PNUE_*A*net_	18.6 ± 1.05 a^*^	13.8 ± 1.04 b	10 ± 1.08 c
PNUE_*V*cmaz_	19.5 ± 0.93 ab	18.2 ± 0.93 b	21 ± 1.1 a
PNUE_*A*sat_	22.1 ± 0.62 a	17.5 ± 0.61 b	14 ± 0.73 c

Values (mean± SE) are the average over four replicates, five nitrogen fertilization rates, and three growing seasons during the period of the season when all stand ages co-occur.

* Different lower case letters indicate significant differences (*P*-value <0.05) between stand ages.

## Discussion

We studied changes in *M.×giganteus* leaf-level photosynthesis as the crop aged during the establishment phase of the crop (first three growing seasons), and the potential interactive effects of N fertilization with age. We found that older *M.×giganteus* stands had 19–30% lower photosynthetic rates during the establishment phase of the crop (first three growing seasons). These changes could be related to N limitation as mature plants were 2.5× larger in size and N_a_ was 14–34% lower than in juvenile plants. N fertilization increased leaf N up to 30% in older stands and allowed photosynthetic performance of fertilized mature stands to reach similar values to unfertilized juvenile stands; however, PNUE was higher in juvenile stands in 2016 and 2017. That is, 1-year-old stands had higher photosynthetic rates with the same leaf N content as older stands, suggesting that other factors were limiting photosynthesis in mature stands.

### Mature stands had lower leaf photosynthesis than juvenile stands across a wide range of photosynthesis indices and growing conditions

Our results support our hypothesis (i) Photosynthesis by age: that *M.×giganteus* leaf photosynthesis in 2- and 3-year-old stands would be lower than in 1-year-old (juvenile) stands and mature stands will not differ from each other. We found a 19–30% age-related decline in *A*_net_ in mature stands, which is within the range of other studies: in the cool season perennial grass *Thinopyrum intermedium*, the age-related decline in *A*_net_ was ~25% (Jaikumar *et al.*, 2016); in another *M.×giganteus* study, it was 38–68% (Boersma *et al.*, 2015), and in pine trees (*Picea abies* and *Pinus sylvestris*) it was ~40% (Niinemets, 2002). Similarly, the age-related decline on ΦPSII (11–33%) is also similar to another *M.×giganteus* study (Boersma *et al.*, 2015). Our study complements previous research on age-related changes in leaf photosynthesis in perennial grasses (e.g. Boersma *et al.*, 2015; Jaikumar *et al.*, 2016) by quantifying age effects on a wider range of leaf photosynthetic parameters using *A*–*C*_i_ and *A*–*Q* response curves. Parameters such as *V*_cmax_ and *A*_sat_ are commonly used in terrestrial biosphere models and could help modelers include age-related changes in their models. We found that age-related effects are larger on CO_2_- and light-saturated *A*_net_ than any of the other parameters of the curves. Juvenile *M.×giganteus* stands had 10–34% higher *V*_cmax_ and *A*_sat_, which suggests that the *A*_net_ differences were not driven by CO_2_ or light limitations.

### Mature stands showed a biphasic dynamic on *A*_net_ that did correspond to other autumnal leaf senescence changes in ϕPSII or leaf nitrogen

Among the mature stands, 2- and 3-year-old stands showed a similar *A*_net_ pattern during the growing season; a constant phase where *A*_net_ varied along a constant mean and a declining phase where *A*_net_ steadily declined until a hard frost. The observed bi-phasic trend is a novel finding for *M.×giganteus*; however, late-season photosynthesis decline occurred in other perennial grasses (Boersma *et al.*, 2015; De Souza *et al.*, 2018; Stavridou *et al.*, 2020; Kar *et al.*, 2020). This biphasic dynamic could be partly driven by temperature differences during the season, as temperature-corrected *A*_net_ did not show this dynamic. However, temperature-corrected observations still showed a steadily decline, suggesting that other factors continue to limit photosynthesis during the growing season. Both ϕPSII and N_a_ showed a steady decline throughout the growing season (Figs 3B, 5), relating this decline to autumnal leaf senescence (Boersma *et al.*, 2015).

### Mature stands had lower leaf N and, while fertilized plants had higher leaf N and photosynthetic rates, N fertilization did not fully compensate for age-related *A*_net_ decline

These differences between stand ages could be driven by N limitation. Mature stands had almost 2.5× more biomass than juvenile stands (Tejera *et al.*, 2019), then for a finite N supply from the soil, N would need to be distributed in a larger volume of biomass in the larger plants, leading to less N per unit of biomass. Older larger plants have larger N reserve pools in the rhizomes, and a larger root system that could potentially compensate the N limitation. We found that N_a_ was 14–34% lower in mature plants than in juvenile plants, supporting hypothesis (ii) Leaf N by age, and did not differ between 2- and 3-year-old stands (Fig. 5). This suggests that the increase in biomass of mature plants relative to juvenile plants is greater than the corresponding increase in leaf N of the youngest ligulated leaf. Previous research shows that N requirements are larger late in the season when plants are bigger (Zapater *et al.*, 2017); here we show this increased N requirement with size also occurs at the same time during the growing season, when stands are of different ages. Given the tight correlation between *A*_net_ and N_a_ (Sinclair and Horie, 1989; Sage *et al.*, 2013; Walker *et al.*, 2014), the decline in leaf photosynthesis in mature stands could be a consequence of the corresponding decline in N_a_.

Both N_a_ and *A*_net_ positively responded to N fertilization, supporting our hypothesis (iii) Photosynthesis by leaf N. N fertilization increased N_a_ by ~30% in fertilized stands over stand ages and years (Table 2); however, N fertilization effect on leaf photosynthesis was not as large and was more variable; its effect increased *A*_net_ by 8%, *V*_cmax_ by 27%, and *A*_sat_ by 14% (Table 2). The disproportional leaf N response to N fertilization compared with the leaf photosynthesis response is rather common in *M.×giganteus*; 4-year-old fertilized *M.×giganteus* had 15% higher N_a_, but had similar *A*_net_ compared with unfertilized plants (Wang *et al.*, 2012). Similarly, in two greenhouse-grown studies, 1-year-old fertilized *M.×giganteus* had ~2× and 2.5× higher N_a_ than unfertilized plants, while their *A*_sat_ was only 20% and 60% higher, respectively (Feng *et al.*, 2012; Ma *et al.*, 2017).

Our experimental design allowed a direct test for the role of N dilution on perennial grass aging: fertilized mature stands against unfertilized juveniles. Fertilized *M.×giganteus* mature stands had similar, or even higher, N_a_ values than unfertilized juvenile stands (Fig. 6), supporting our hypothesis (iv) Age by N fertilization. However, the N fertilization effect on leaf photosynthesis of mature *M.×giganteus* stands was not as large and was more variable. While N fertilization increased leaf photosynthesis, it did not completely close the age gap. N fertilization reduced the age decline on *A*_net_ and ϕPSII by ~50% and actually stimulated an increase in *V*_cmax_ levels ~10% greater than those seen in juveniles (Fig. 6; Supplementary Fig. S5). Ultimately, a comparison of leaf photosynthesis per unit of N (i.e. NUE) among stand ages controls much of the variability and shows that juvenile stands had at least 26% higher PNUE_*A*net_ or PNUE_*A*sat_ than mature stands across all N fertilization rates (Fig. 7). This implies that while N fertilization increased leaf photosynthesis in mature stands, in some cases up to juvenile levels, this increment was largely driven by a disproportionally larger change in leaf N, which could indicate a luxury consumption.

### Late-season sink limitations could drive the mature stands’ decline in photosynthesis

If N fertilization alone did not compensate for the age decline in photosynthesis, what other factors could limit photosynthesis in mature stands, and not as strongly limit photosynthesis in juvenile plants? We found that temperature was an important driver of the bi-phasic dynamic; however, temperature-corrected *A*_net_ still showed a steady decline over the growing season, suggesting that other factors may also limit photosynthesis during the season. The current understanding of temperature correction assumes that temperature is the only limiting factor. This assumption is valid when changes in CO_2_ assimilation are studied over a range of sequential temperatures (e.g. temperature response curves). It is worth pointing out that in the field setting when changes in temperature occur during the growing season, they involve changes in development and reserve status. We hypothesize that sink limitations may play an important role in the photosynthesis of mature stands of perennial grasses. Sink limitations occur when the main carbohydrate sinks (e.g. rhizomes and growing points) have ceased their activity, and recently assimilated carbohydrates are consumed at a lower rate than they are produced. Ultimately, carbohydrates accumulate in the leaf, trigger feedback limitations on photosynthesis, and reduce *A*_net_. Sink limitations have already been suggested to limit photosynthesis and late-season growth of perennial grasses, (McCormick *et al*., 2006, 2009). Here we propose that sink limitations could help explain the photosynthetic decline observed in mature stands late in the season, and the photosynthetic decline observed in mature stands compared with juveniles.

From Tejera *et al.* (2021), we know that mature *M.×giganteus* plants reached a steady number of tillers in mid-June, and completed vegetative development by the end of August. Since growth represents a major sink of carbohydrates, mature plants could experience sink limitations around late summer when vegetative development had ceased. Accordingly, mature stands showed that *A*_net_ started declining around the same time; early September in 2016 and late July in 2017. While this marked declined was not observed in temperature-corrected *A*_net_, it still showed a steady decline, suggesting other limiting factors. Altogether this suggests that the *A*_net_ decline observed in mature stands late in the season could be related to sink limitations driven by reduced growth. When comparing stand ages, growth may remain active, both above- and below-ground, in juvenile stands for longer in the season. Juvenile stands did not reach reproductive stages in 2016 or 2017, and continued to grow until the late autumn. In turn, mature stands ceased vegetative growth and transition to reproductive growth much earlier in the growing season (Tejera *et al.*, 2021). In addition, below-ground biomass is known to increase in juvenile stands and remains constant after the third year of growth, when plants reached the mature phase (Nassi o Di Nasso *et al.*, 2013). Considering that *M.×giganteus* does not produce seeds and biomass allocation to reproduction is minimal, older stands by the end of the growing season may lack major sinks to which to direct photosynthates, ultimately limiting photosynthesis, leading to the observed photosynthetic decrease in mature stands.

Complementing leaf photosynthesis and N seasonal observations with seasonal sampling of carbohydrate reserves would help test this hypothesis. In sugarcane, leaf photosynthesis steadily declined during the growing season, while leaf sucrose and starch, and root sucrose reached a maximum concentration towards the end of development, suggesting a sink limitation late in the season when plants ceased growth (De Souza *et al.*, 2018). It is worth highlighting that, while leaf-level photosynthesis was lower in older plants, on the whole they were bigger and have more leaves (Tejera *et al*., 2019, 2021); therefore, they would still have greater canopy-level carbon assimilation.

*Miscanthus* spp. breeding programs usually focus on traits such as later flowering to extend the vegetative growth season and achieve higher yields. Here, we suggest that prolonging vegetative growth could keep sink organs active, maintaining higher photosynthesis for longer in the season. In addition, breeding for genotypes that are more tolerant to sink limitations could be a successful strategy to maintain higher rates of photosynthesis for longer in the season and reduce the yield decline after peak yields.

### Conclusion

The study of age-related changes in perennial physiology includes many confounding factors that limit our understanding of the underlying mechanisms. We add to previous research on the role of micro-environmental conditions on aging (e.g. Vitasse, 2013), by directly testing whether age-related changes in photosynthesis are due to N dilution in larger older plants under field conditions. Our staggered-start experimental design allowed us to conduct this work in the field while also controlling for stochastic and environmental conditions. Mature stands had lower photosynthetic rates and leaf N contents, suggesting potential N dilution in older larger plants. However, N fertilization increased mature *Miscanthus* N_a_ up to the levels of juveniles, but the photosynthetic response per unit of N was much lower, suggesting that N was not a limiting factor. Considering that mature stands cease growth much sooner in the growing season than juvenile stands, and that juvenile stands allocate more biomass to below-ground organs, we hypothesize that sink limitations could drive the age-related difference between juvenile and mature *M.×giganteus* plants. More research is needed to understand how leaf photosynthesis scales to canopy-level photosynthesis in *M.×giganteus*, and whether this age-related change holds at the canopy level.

## Supplementary data

The following supplementary data are available at *JXB* online.

Table S1. Parameters of net CO_2_ assimilation response to light response curves.

Table S2. Parameters of net CO_2_ assimilation response to internal CO_2_ concentration response curves.

Fig. S1. Leaf temperature distribution and net CO_2_ assimilation response to leaf temperature.

Fig. S2. Temperature-corrected net CO_2_ assimilation over the growing season.

Fig. S3. Temperature-corrected maximum carboxylation rate of Rubisco (*V*_cmax_) and light-saturated net CO_2_ assimilation (*A*_sat_).

Fig. S4. Specific leaf area over the growing season.

Fig. S5. Nitrogen effect on leaf CO_2_ assimilation, PSII efficiency, and leaf nitrogen age decline.

erac382_suppl_Supplementary_Material

## Data Availability

The datasets generated and/or analyzed during the current study are available in the Mendeley Data repository at https://data.mendeley.com/datasets/tr37gtnjmy (Tejera and Heaton, 2022).

## Abbreviations

*A*_net_: leaf photosynthetic net CO_2_ assimilation
*A*_sat_: light-saturated *A*_net_
N: nitrogen
N_a_: leaf N mass per unit leaf area
PNUE: photosynthetic nitrogen use
ϕPSII: PSII efficiency
*R*_d_: leaf respiration in the dark
*V*_cmax_: maximum carboxylation rate of Rubiscwo
*V*_pmax_: maximum PEP carboxylation rate
Φ: maximum quantum yield
