# Supplementary material for: Photosynthetic decline in aging perennial grass is not fully explained by leaf nitrogen
Source: J Exp Bot. 2022 Oct 4;73(22):7582–95. doi: 10.1093/jxb/erac382 (PMC9730795; doi:10.1093/jxb/erac382)
Supplement: erac382_suppl_Supplementary_Material [file erac382_suppl_supplementary_material.pdf]

Supplementary Information: Photosynthetic decline in aging perennial grass is not fully explained by leaf nitrogen

Mauricio Tejera[1,2], Nicholas N. Boersma[2,3], Sotirios V. Archontoulis[2], Fernando E. Miguez[2], Andy VanLoocke[2,3], Emily A. Heaton[2,3,4*]

1 Great Lakes Bioenergy Research Center, Michigan State University, East Lansing, MI, USA.

2 Department of Agronomy, Iowa State University, Ames, IA, USA

3 Center for Advanced Bioenergy and Bioproducts Innovation, Urbana, IL, USA

4 Crop Sciences, University of Illinois, Urbana-Champaign, IL, USA

* Corresponding author (heaton6@illinois.edu)

Table S1: A – Q curve parameters

Table S1: Unfertilized *Miscanthus* × *giganteus* *A* – Q curve parameters (mean ± s. e.) as affected by stand age and sampling date in 2015, 2016 and 2017. Different letters indicate significant differences between sampling dates and stand ages, within years (P < 0.05).

	A_{sat} (µmol CO_2 m^{-2} s^{-1})	QEff	Theta	Rd
2015 - 1 year (yr) old				
Aug, 11	39.7 ± 1.72 a	0.1 ± 0	2.9 ± 0.24 ab	0.6 ± 0.11
Aug, 26	41.2 ± 1.98 a	0.1 ± 0	2.5 ± 0.26 b	0.5 ± 0.13
Sep, 09	33.7 ± 1.31 b	0.1 ± 0	3.7 ± 0.27 a	0.7 ± 0.08
Oct, 17	15 ± 1.87 c	0.1 ± 0.03	1.1 ± 0.4 c	-0.9 ±.75
Nov, 06	9.5 ± 1.28 c	0.1 ± 0.08	0.7 ± 0.47 c	-1.3 ± 4.52
2016				
Jun, 06				
2 yr old	28 ± 1.63 1	0.047 ± 0.0035 b	0.9 ± 0.047 a	2.3 ± 0.37 b
Jul, 14				
2 yr old	39.2 ± 2.28 2	0.066 ± 0.007 a	0.5 ± 0.207 b	3 ± 0.41 ab
Sep, 20				
1 yr old	45.2 ± 1.28 3	0.061 ± 0.0012 b	0.8 ± 0.016 a	5 ± 0.36 a
2 yr old	35.3 ± 1.28 2	0.063 ± 0.0018 b	0.7 ± 0.031 ab	3.9 ± 0.36 ab
Oct, 11				
1 yr old	35.1 ± 1.81 2	0.059 ± 0.0021 b	0.7 ± 0.038 ab	2.7 ± 0.5 ab
2 yr old	26.7 ± 1.78 1	0.063 ± 0.0032 b	0.6 ± 0.054 b	1.9 ± 0.51 b
2017				
Jun, 07				
2 yr old	42.6 ± 2 a	0.064 ± 0.00229 a	0.7 ± 0.04 ab	3.5 ± 0.19 a
3 yr old	34.7 ± 1.6 ab	0.061 ± 0.00185 ab	0.7 ± 0.032 ab	3.3 ± 0.16 a
Jul, 25				
2 yr old	37.2 ± 1.85 ab	0.061 ± 0.00205 ab	0.8 ± 0.032 a	3.8 ± 0.18 a
3 yr old	38.3 ± 1.61 a	0.057 ± 0.00157 ab	0.8 ± 0.025 a	3.3 ± 0.15 a
Sep, 11				
1 yr old	34.8 ± 1.2 ab	0.058 ± 0.00178 bc	0.8 ± 0.03 ab	3.3 ± 0.24 a
2 yr old	29.3 ± 1.34 bcd	0.052 ± 0.00179 ab	0.8 ± 0.024 a	3.3 ± 0.28 a
3 yr old	29.6 ± 1.16 bc	0.056 ± 0.00162 abc	0.8 ± 0.02 b	2.9 ± 0.24 a
Sep, 27				
1 yr old	36.3 ± 1.27 b	0.063 ± 0.00233 a	0.6 ± 0.054 b	3.4 ± 0.25 a
2 yr old	26.2 ± 1.41 bcd	0.06 ± 0.00288 abc	0.8 ± 0.04 ab	2.8 ± 0.3 ab
3 yr old	23.7 ± 1.25 def	0.06 ± 0.00299 bc	0.6 ± 0.061 b	1.8 ± 0.26 bc
Oct, 16				
1 yr old	29.1 ± 1.26 cd	0.049 ± 0.00214 a	0.6 ± 0.06 b	2.5 ± 0.24 abc
2 yr old	22.8 ± 1.36 ef	0.054 ± 0.00298 abc	0.7 ± 0.063 ab	1.6 ± 0.29 bc
3 yr old	20.4 ± 1.16 f	0.054 ± 0.00275 abc	0.7 ± 0.054 ab	1.4 ± 0.25 c

Table S2: A – C_i curve parameters

Table 2: Unfertilized *Miscanthus* × *giganteus* *A* – Ci curve parameters (mean ± s. e.) as affected by stand age and sampling date in 2015, 2016 and 2017, according to an asymptotic exponential regression. Different letters indicate significant differences between sampling dates and stand ages, within years (P < 0.05)

	A_{sat} (μmol CO_2 m^{-2} s^{-1})	lrc (μmol CO_2 m^{-2} s^{-1} (μmol m^{-2} s^{-1})$^{-1}$)	c0
2015 - 1 year (yr) old			
Aug, 11	41.9 ± 1.11 a	-4.4 ± 0.03 a	8.10 ± 2.80 b
Aug, 26	37.1 ± 1.03 b	-4.5 ± 0.03 a	7.60 ± 2.64 b
Sep, 09	34.9 ± 1.08 b	-4.7 ± 0.05 b	25.1 ± 3.01 a
Oct, 17	8.20 ± 1.06 c	-4.3 ± 0.20 ab	24.4 ± 7.01 ab
Nov, 06	8.90 ± 1.58 c	-4.9 ± 0.31 ab	25.8 ± 14.2 ab
2016			
Jun, 06			
2 yr old	38.9 ± 1.07 a	-4.5 ± 0.08	15 ± 2.28 b
Jul, 14			
2 yr old	31.8 ± 1.12 b	-4.4 ± 0.11	24.9 ± 2.91b
Sep, 20			
1 yr old	43.4 ± 1.45 a	-4.3 ± 0.08	24.4 ± 2.06 b
2 yr old	31.9 ± 1.29 b	-4.1 ± 0.10	34.0 ± 2.29 a
Oct, 11			
1 yr old	26.5 ± 1.85 bc	-4.2 ± 0.22	17.3 ± 4.5 b
2 yr old	23.8 ± 1.47 c	-4.0 ± 0.15	15.1 ± 3.2 b
2017			
Jun, 07			
2 yr old	38.2 ± 1.66 a	-4.9 ± 0.15 b	9.4 ± 8.68 b
3 yr old	39.3 ± 1.45 a	-4.9 ± 0.11 b	30.3 ± 5.8 b
Jul, 25			
2 yr old	41.1 ± 2.00 a	-4.8 ± 0.16 b	-1.7 ± b0.53 b
3 yr old	35.3 ± 1.32 a	-4.4 ± 0.14 a	12.9 ± 5.29 b
Sep, 11			
1 yr old	38.3 ± 1.55 a	-4.9 ± 0.1 b	4.8 ± 7.85 b
2 yr old	29.9 ± 1.77 bc	-4.9 ± 0.13 b	30 ± 8.63 b
3 yr old	33.3 ± 1.63 ab	-4.8 ± 0.12 b	22.8 ± 8.16 b
Sep, 27			
1 yr old	30.3 ± 1.32 bc	-4.3 ± 0.1 a	52.7 ± 5.98 a
2 yr old	29.7 ± 1.64 bc	-4.6 ± 0.13 b	30.9 ± 8.56 b
3 yr old	22.3 ± 1.30 d	-4.3 ± 0.13 a	55.5 ± 6.65 a
Oct, 16			
1 yr old	28.0 ± 1.35 cbd	-4.8 ± 0.12 b	14.6 ± 8.43 b
2 yr old	25.5 ± 1.58 cd	-4.9 ± 0.15 b	21.3 ± b0.43 b
3 yr old	25.4 ± 1.62 cd	-5.2 ± 0.16 b	-6.9 ± b6.42 b

Figure S1: Leaf temperature distribution and net CO_2 assimilation response to leaf temperature

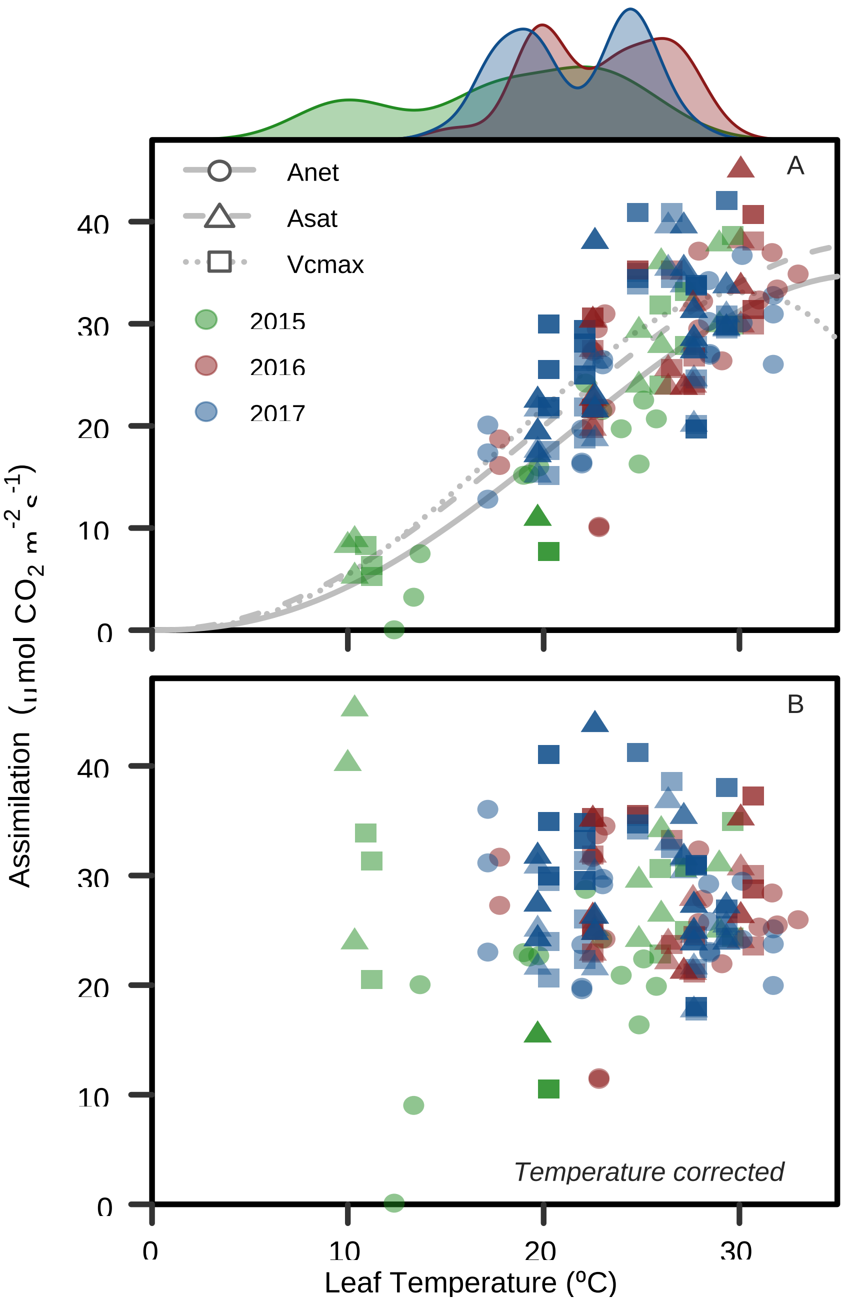

Figure S1: Leaf temperature effects on *Miscanthus x giganteus* net CO_2 assimilation. (A) Response of net CO_2 assimilation at ambient conditions (A_{net}), and CO_2 and light saturated conditions (V_{cmax} and A_{sat}, respectively) to leaf temperature. Density plots on top of A show leaf temperature distribution in 2015, 2016 and 2017. (B) Temperature-corrected A_{net}, V_{cmax} and A_{sat} response to temperature. Temperature corrections were performed based on Bernacchi et al, (2003), see manuscript for details on the calculation.

Figure S2: Temperature-corrected net CO_2 assimilation over the growing season

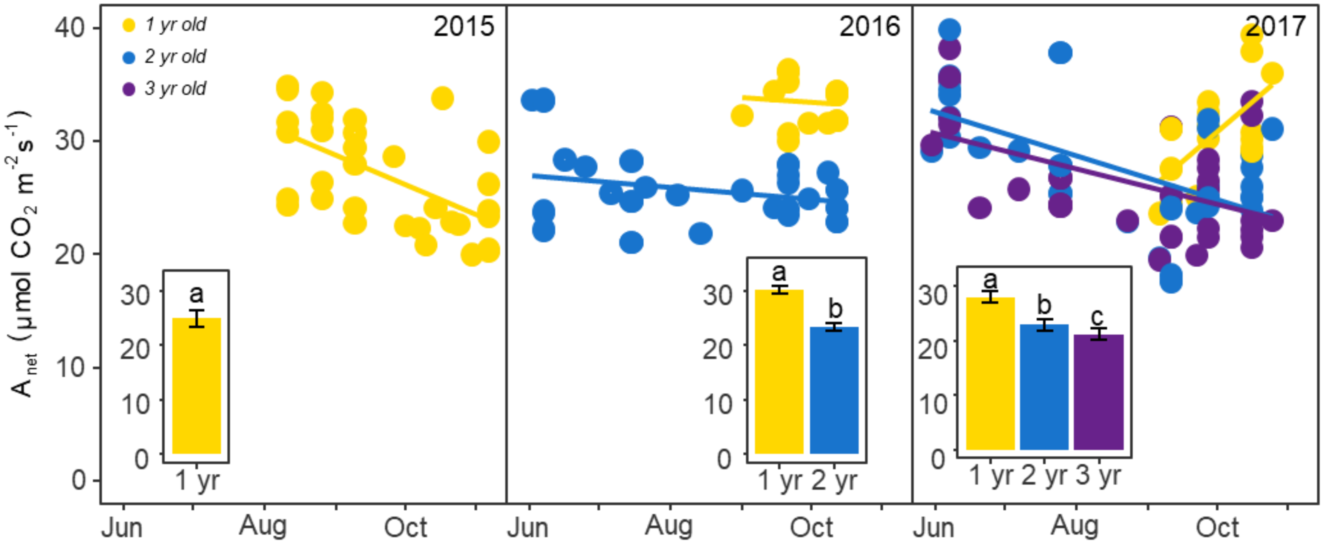

Figure S2: Changes in *Miscanthus x giganteus* in temperature-corrected net CO_2 assimilation (A_{net}) at different stand ages along 2015, 2016 and 2017 growing seasons. Data are from unfertilized plots averaged over 4 repetitions. Error bars correspond to ± one standard error of the mean. Inset graphs show average temperature corrected A_{net} over the period of the year when all stand ages occur at the same time. Different letters indicate significant differences between stand ages at p-value < 0.05.

Figure S3: Temperature-corrected maximum carboxylation rate of rubisco (V_{cmax}) and light saturated net CO 2 assimilation (A_{sat})

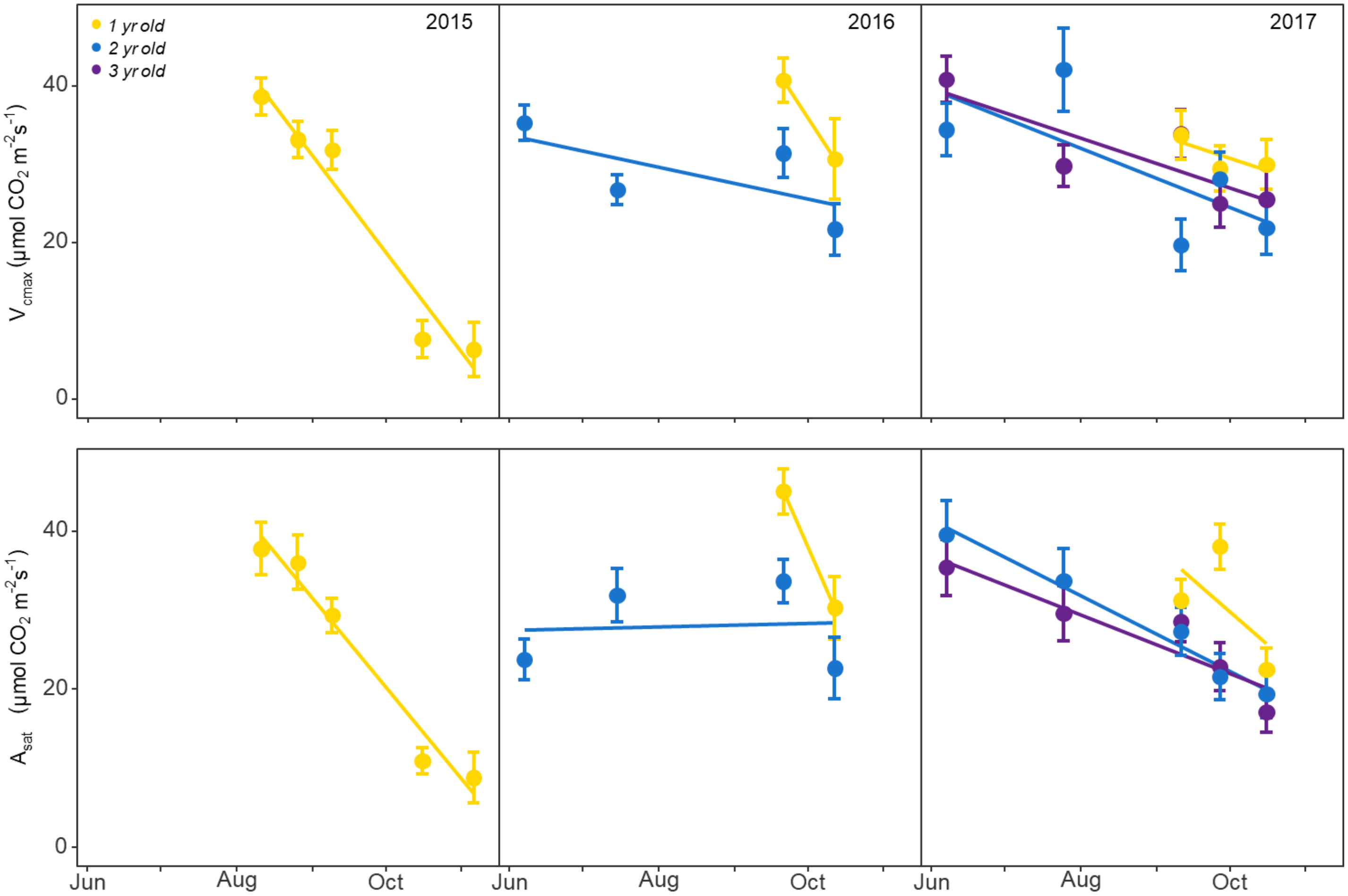

Fig. S3: Changes in *Miscanthus* × *giganteus* temperature-corrected maximum carboxylation rate of rubisco (V_{cmax}; top row) and light saturated net CO_2 assimilation (A_{sat}; bottom row) at different stand ages during the 2015, 2016 and 2017 growing seasons. Data are averages from unfertilized plots (n=4). Error bars correspond to ± one standard error of the mean. Inset graphs compile all net CO_2 assimilation response curves to internal CO2 concentration (Ci) and PPFD during the period of the year when all stand ages occurred at the same time.

Figure S4: Specific Leaf Area over the growing season

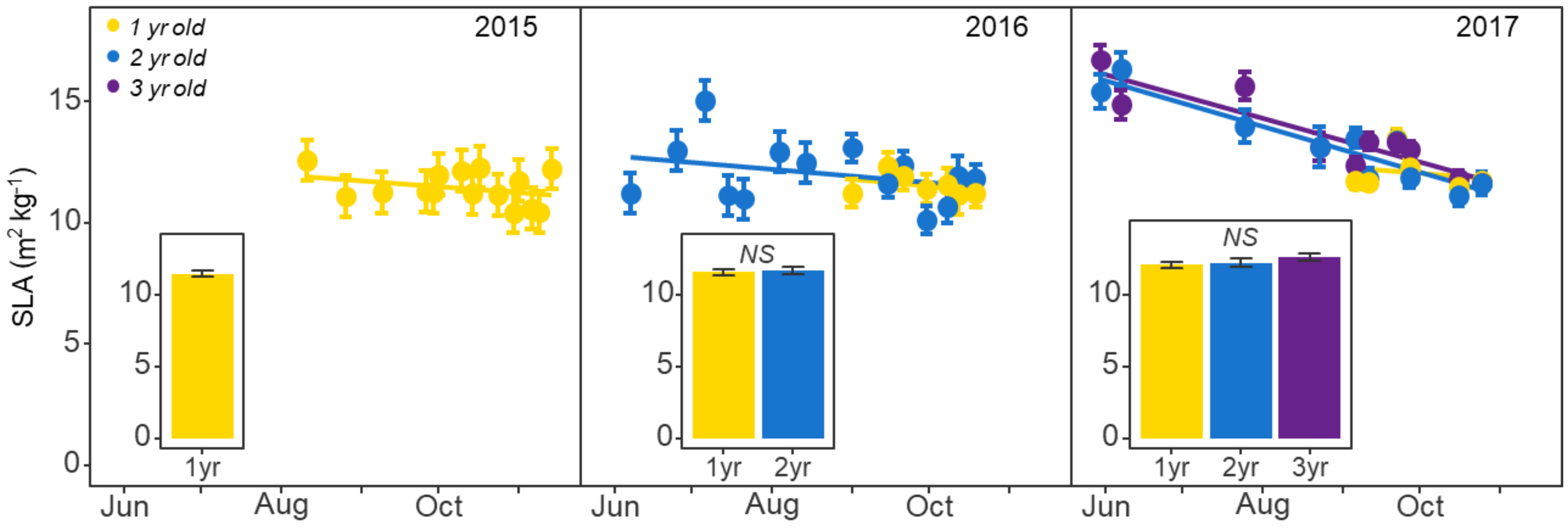

Fig. S4: Age effects on *Miscanthus x giganteus* specific leaf area (SLA) of the youngest ligulated leaf in 2015, 2016 and 2017 (left, center and right panel, respectively). Data points are averaged over 4 blocks. Inset graphs show average SLA over the period of the year when all stand ages occurred at the same time. NS indicates significant no differences between stand ages at p-value < 0.05. Error bars show ± one standard errors of the mean.

Figure S5: Nitrogen effect on age-related photosynthetic decline

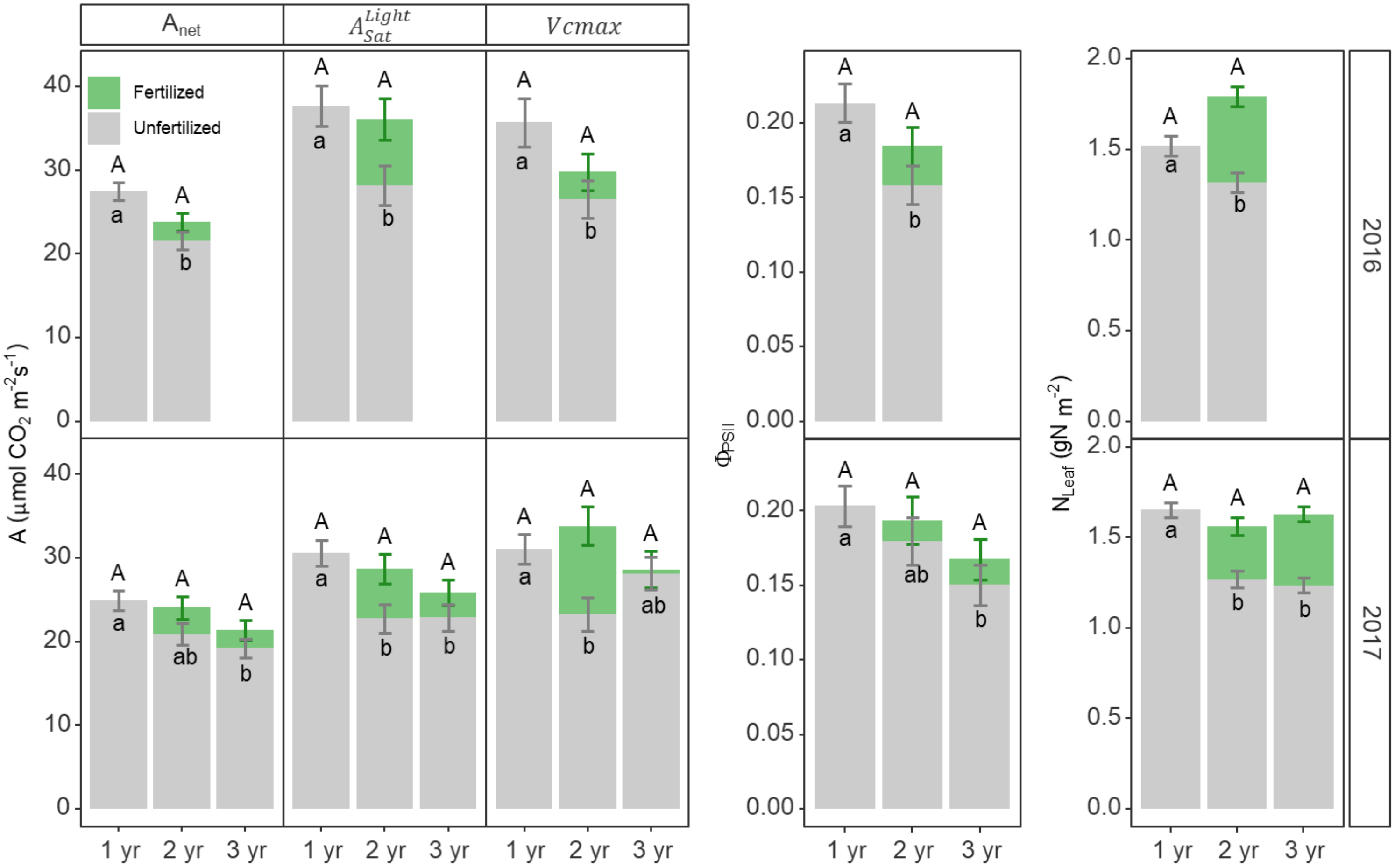

Fig. S5: Nitrogen (N) fertilization effects on *Miscanthus x giganteus* age-related changes in leaf photosynthetic parameters. Left: net co2 assimilation (A_{net}), maximum carboxylation rate of rubisco (Vcmax) and light saturated A_{net} (A_{Sat}^{Light}) Right: photosystem II efficiency ($\phi PSII$) and area-based leaf nitrogen content (N_a) during the period when all stand ages cooccurred (Sept – Nov). Different lower-case letters indicate significant differences between unfertilized stand ages (grey bars; $P < 0.05$). Different upper-case letters indicate significant differences between unfertilized juvenile (one-year-old) and fertilized mature (two- and three-year-old; green bars; $P < 0.05$). Values are the average of four blocks, error bars show ± one standard errors of the mean.
